# Do difficulties with emotion regulation influence the empathic skills of Tunisian psychiatry trainees?

**DOI:** 10.1192/j.eurpsy.2025.1646

**Published:** 2025-08-26

**Authors:** Y. Trabelsi, B. N. Saguem, Z. Bouzaabia, J. Nakhli

**Affiliations:** 1 Psychiatry Department, Farhat Hached Hospital, sousse, Tunisia

## Abstract

**Introduction:**

Empathy is essential in psychiatry, and emotion regulation significantly influences it. This study investigates the link between emotion regulation difficulties and empathic abilities in Tunisian psychiatry trainees.

**Objectives:**

This study aimed to explore the connection between empathic abilities and challenges in emotion regulation among Tunisian psychiatry trainees.

**Methods:**

A cross-sectional study was carried out involving an online survey proposed to 120 Tunisian psychiatry trainees. The survey included the Interpersonal Reactivity Index, which features four subscales: Perspective Taking (PT), Empathic Concern (EC), Personal Distress (PD), and Fantasy Scale (FS). A cut-off score of 14 was set to differentiate between low and high empathy levels for each subscale. Additionally, the survey included the Difficulties in Emotion Regulation Scale (DERS), which evaluates six types of emotion regulation challenges.

**Results:**

The proportion of responses was 71% and the mean empathy subscores were: (19.04 ± 3.95) for PT, (20.41 ± 3.71) for EC, (12.67 ± 4.41) for PD and (16.40 ± 4.91) for FS. Eleven participants showed low levels of PT and 30 showed high scores of PD. Empathy dimensions’ scores, mainly those of PT and PD, were correlated with ER difficulties. PT scores were negatively correlated with five ER difficulties and PD scores were positively correlated with six ER difficulties of the DERS.

Lower levels of PT were associated to difficulties engaging in goal-directed behaviors (p=0.038) and impulse control difficulties (p=0.004) furthermore higher scores of PD were associated to difficulties engaging in goal-directed behaviors (p=10^-3^), impulse control difficulties (p=10^-3^), strategy (p=0.003), non-acceptance (p=0.001) and clarity (p=0.001).

**Conclusions:**

Difficulties with emotion regulation were positively associated with personal distress and negatively associated with perspective-taking abilities. Our findings highlight the significance of emotion regulation processes in improving empathic skills.

**Disclosure of Interest:**

None Declared

